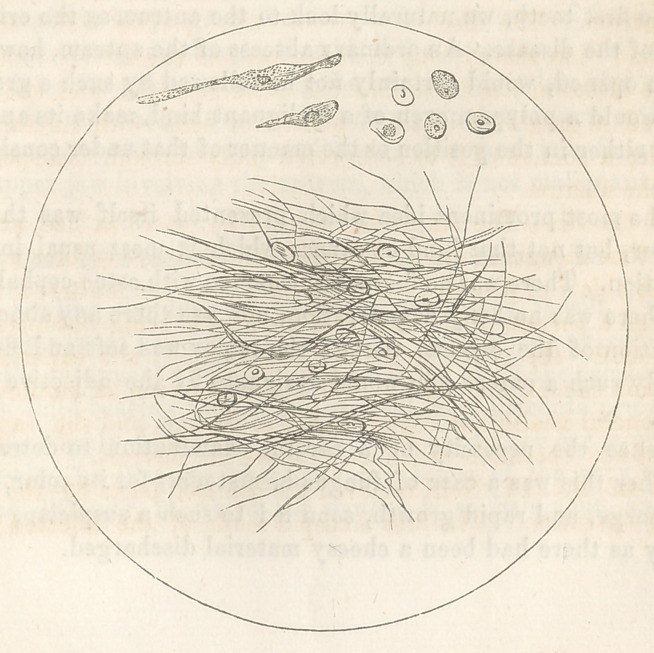# Fungus of the Upper Jaw and Its Successful Removal

**Published:** 1854-04

**Authors:** John Neill

**Affiliations:** Surgeon to the Pennsylvania Hospital


*Fungus of the Upper Jaw and its Successful Removal.* By JOHN NEILL, M. D., Surgeon to the Pennsylvania Hospital.

From the Notes of Dr. RHOADS, House Surgeon.

To those who are familiar with tumors of the mouth and face, the title of this paper will probably convey an erroneous or unsatisfactory impression, and yet we are ignorant of any other term which can be more correctly used.

It would naturally be supposed that this was a case of fungus hæmatodes or medullary fungus, and that we mean to imply that this was a malignant disease usually designated by this generic term. In order to express the sense, however, in which the term is used, and to give a clear account of the affection, it will be necessary to give a history of the case, and then compare it with other affections, so that its true nature may be understood.

"Mary Fitzpatrick, aged 18, was admitted 5th mo. 19, 1853, under care of Dr. Neill.

She is of medium size, has a fine rosy complexion and an appearance of general good health, which she has always enjoyed, with the exception of some imperfection of vision for which she was treated three months since at the Wills' Hospital, and from which she has entirely recovered.

She states that eight weeks since, she first perceived a swelling upon the inner side of the last molar tooth of the upper jaw upon the left side, which she at first supposed to be a gum boil, but as it grew large she consulted a physician, who lanced the swelling. Almost immediately a fungous growth sprouted from the incision, the tooth became loose and was extracted, and the disease extending rapidly, she was induced to apply to the Hospital for relief.

On admission, the left cheek was slightly swollen and tender immediately beneath the malar prominence; and a soft fungus, the size of a hickory nut, was found projecting into the cavity of the mouth from the spot whence the tooth had been extracted.

The surrounding parts were much swollen, the tooth in front of the one which had been lost being almost covered by the spongy gum and also moveable in its socket. The fungus was of a dark reddish hue, except on the under surface, which appeared to be sloughing, and had an ash-grey color.

A probe passed readily between the fungus and the tooth into the antrum. The discharge through this opening, which at one time had been cheesy, was thin, sanious and excessively fetid.

The loosened tooth was directed to be removed, and she was ordered a gargle of diluted chloride of soda with tincture of myrrh; a portion of it to be injected daily through the opening into the antrum.

*5th mo.* 31.—The fungus has rapidly increased in size, at the same time sloughing upon its surface; the distressing fetor makes her an object of disgust even to herself; the voice is altered, from so large a portion of the cavity of the mouth being occupied by the tumor; the cheek is much more swollen; she experiences constant nausea, and rejects food almost as soon as swallowed; her general health has begun visibly to decline, and she eagerly desires any operation which may relieve her distress.

*6th mo.* 2.—Dr. Neill removed the diseased parts by the following operation. The first bicuspid tooth of the affected side having been extracted, an incision was carried through the structures of the cheek in a nearly horizontal line from the corner of the mouth beyond the anterior edge of the masseter muscle, and in such a manner as to avoid the duct of Steno.

The upper flap being raised, the alveolar process was divided with the bone forceps at the point whence the tooth had been extracted for the purpose.

Then with the curved bone forceps of Liston, the exterior wall of the antrum was divided through its whole extent, and next the palate process of the superior maxillary bone; care being taken to remove as much as possible. The soft parts were now severed by the scissors from the posterior edge of the bony palate, and the mass shaken loose, when the division of a few remaining fleshy attachments completed its separation.

The cavity of the antrum was found filled with the fungus which, however, was attached only to the floor, leaving the lining membrane of the remainder of the antrum healthy.

The hemorrhage was moderate; a few arteries required ligatures, and the parts having been well freed from coagula, the cut edges of the cheek were approximated by the twisted suture, strips and collodion.

The tumor was found to originate from the bone; the tissues of the two being intimately blended. The central part was of a pale straw color, and jelly-like appearance, but tough. The fungus was friable, and dark red from effused blood. Under the microscope the tissue of the tumor presented fibro-plastic nuclei, fibre cells and fibres.

*6th mo.* 4.—Some febrile reaction followed the operation, but has now subsided. The pins were removed to-day; the cut surfaces adhere, and are well supported by the strips and collodion.

*6th mo.* 30.—She was discharged, cured. The wound in the cheek has entirely healed, both within and without, leaving the least possible scar on the latter surface."

*Remarks.*—When this patient opened her mouth and this rounded tumor was seen projecting from the gum, the idea that it was epulis first suggested itself. Epulis, however, though it may occur in this situation, does not possess similar characters; it is a fibrous structure—a simple growth emanating from the gum or periosteum; it is of a firm consistence, and, when small, is covered by a smooth membrane. It commences by a small, seed-like, warty excresence, grows very slowly, without pain, and is usually of the color of the gum, or sometimes somewhat lighter.

Moreover, when it is observed that this growth sprouted rapidly from an incision supposed to have been made into a gum-boil, and that the cavity of the antrum was opened by the extraction of the first tooth, we naturally look to the antrum as the original seat of the disease. An ordinary abscess of the antrum, however, when opened, would certainly not be followed by such a growth, nor would a polypus, even of a malignant kind, make its appearance either in the position or the manner of that under consideration.

The most prominent idea which presented itself was that of cancer, but not that kind of cancer which is most usual in this situation. There was no feature in common with osteo-cephaloma, for there was no bony degeneration, nor was there any abnormal nutrition of the osseous tissue; the tumor was soft and fleshy, simply such a one as you would designate by the adjective *fungous.*

Hence the necessity of a careful examination to determine whether this was a case of fungus hæmatodes; for its color, fetid discharge, and rapid growth, soon led to such a suspicion, especially as there had been a cheesy material discharged.

The antrum is frequently the seat of medullary cancer, and it is quite likely to occur in early life. According to Liston, it originates at the root of a tooth, or from the membrane lining the maxillary sinus; it grows rapidly, produces pain, bursts the cavity, discharges a soft, brain-like material, and blood and sanies; the teeth are loosened and a fungus rapidly grows.

With such a view, no time was to be lost; the tumor was growing rapidly, and the only chance of its cure was based upon its thorough removal, and the hope that it might not prove to be what it seemed.

The operation was commenced with the intention of removing the whole of the superior maxillary bone, if it were necessary, but the sides and floor of the antrum, with a portion of the roof of the mouth being removed as a preliminary operation, the interior of the antrum could then be satisfactorily exposed, and the fungus being found not to involve the upper and posterior walls, they were allowed to remain.

The microscopic examination of the mass after the operation, gave satisfactory evidence of the benign character of the affection; and the present sound condition of the patient corroborates this opinion and justifies the course pursued; and we, therefore, are disposed to consider this case as a similar one to that which Mr. Liston describes when speaking of epulis, "A soft tumor of the gum, rapid in its progress, broken on its surface, and furnishing fetid and bloody discharge, is said to be sometimes met with; there is no danger of mistaking the one for the other, the remediable for the malignant, and fortunately the latter is rare;" and also to agree with Jourdain and others that there is a fungus of the upper jaw involving the antrum, which is not malignant.

## Figures and Tables

**Figure f1:**